# Chemical Drivers of Flavor Variation Across Cultivars and Grades of Fujian White Tea Revealed by Integrated Volatile and Non-Volatile Metabolomics

**DOI:** 10.3390/foods15030458

**Published:** 2026-01-28

**Authors:** Fuli Zong, Zi Yang, Linping Xiao, Yan Tong, Lan Shen, Zhijie Dong, Jianwei Zhou, Huan Cheng, Wenjun Wang, Donghong Liu

**Affiliations:** 1 College of Biosystems Engineering and Food Science, National-Local Joint Engineering Research Center of Intelligent Food Technology and Equipment, Zhejiang Key Laboratory of Agro-Food Resources and High-Value Utilization, Fuli Institute of Food Science, Zhejiang University, Hangzhou 310058, China; 2 Institute of Food Science, HongSheng Beverage Group Co., Ltd., Hangzhou 311200, China; 3 Future Food Laboratory, Innovation Center of Yangtze River Delta, Zhejiang University, Jiaxing 314100, China; 4 School of Mechatronics and Energy Engineering, NingboTech University, Ningbo 315100, China

**Keywords:** white tea, metabolomics, volatile compounds, non-volatile metabolites, grade, cultivar

## Abstract

Grade and cultivar are the important factors influencing white tea quality, but their relative metabolic contributions are not fully understood. Twelve white tea samples representing four major Fujian cultivars across three grades were analyzed using UHPLC–MS-based non-volatile metabolomics, HS-SPME–GC–MS volatile profiling, and sensory correlation analysis. In total, 47 non-volatile and 21 volatile markers were associated with grade differences, while 44 non-volatile and 26 volatile markers were linked to cultivar differences. Catechins and amino acids declined as grade decreased, whereas flavonol glycosides and gallic acid increased, accompanied by stronger astringency and reduced umami and sweetness. Aroma profiles showed a similar trend, with higher-grade teas dominated by floral notes and lower-grade teas exhibiting more herbal characteristics. Dimeric catechins, oxylipins, and aroma glycosides varied among cultivars. Volatile profiles separated the cultivars into two aroma groups: Fuding Dabai and Fuding Dahao showed more floral–fruity aromas, whereas Fuan Dabai and Zhenghe Dabai exhibited stronger herbal and aged aromas. Odor activity value analysis showed that linalool, geraniol, and (*E*,*Z*)-3,6-nonadien-1-ol were among the most abundant aroma-active compounds across white tea samples. These results provide chemical evidence for distinguishing white tea by grade and cultivar, with potential relevance to quality evaluation.

## 1. Introduction

Tea (*Camellia sinensis*) possesses a long cultural history and is valued globally as a health-beneficial beverage [1]. From a processing and biochemical standpoint, teas are classified into six major types: green, white, yellow, oolong, black, and dark tea [2]. Among them, white tea undergoes the least processing, typically involving only gentle withering followed by drying [3]. Such minimal handling permits limited enzymatic oxidation during withering, allowing amino acids and polyphenols to be largely retained [4]. These constituents contribute not only to its sweetness and freshness but also to its reported antioxidant and neuroprotective properties [5,6]. In addition to taste modulation, withering constitutes a key stage in aroma formation, as moisture loss promotes the conversion of multiple biochemical precursors into the characteristic floral and herbal volatiles of white tea [7].

Tea flavor emerges from the collective action of multiple chemical classes [6]. Bitterness and astringency are mainly derived from catechins, caffeine, and flavonol glycosides, whereas amino acids contribute to umami and sweetness [8]. However, these attributes do not arise from single metabolites but from the combined effects of non-volatile taste compounds, volatile aroma constituents, and their metabolic precursors [9]. Accordingly, comprehensive profiling approaches are required to describe flavor composition at the system level.

Against this background, cultivar represents a major biological factor underlying variation in white tea flavor composition. Fujian Province is the core production area of white tea. Its unique ecological conditions and long-standing processing techniques have given rise to four widely recognized cultivars: Fuding Dabai (FDDB), Fuding Dahao (FDDH), Zhenghe Dabai (ZHDB), and Fuan Dabai (FADB) [3]. Cultivar strongly influences tea chemistry due to intrinsic differences in nitrogen metabolism, flavonoid pathways, and volatile formation [10]. This biochemical divergence is reflected in distinct aroma-active compounds across cultivars. For example, comparative studies between FADB and ZHDB have identified discriminating odorants such as 1-octen-3-ol, benzyl alcohol, nerol, *β*-ionone, and *δ*-cadinene [11]. Pronounced differences in amino acids, soluble sugars, and other volatiles have likewise been reported among FDDB, FDDH, ZHDB, and FADB, underscoring cultivar as a key contributor to white tea’s chemical and sensory diversity [12].

Leaf maturity represents another fundamental determinant of white tea quality. Differences in tenderness regulate nitrogen-related metabolites, catechin levels, and early oxidative reactions during withering [13]. Tender buds generally contain higher concentrations of free amino acids and soluble proteins. In contrast, mature leaves show a decline in nitrogenous metabolites and monomeric catechins, alongside an increase in flavonol glycosides and phenolic acids, a shift driven by enhanced senescence-related metabolism [14]. These maturity-related chemical patterns remain consistent across cultivars and underlie the graded differences in taste and aroma profiles. Sensory studies further indicate that tender buds and the first leaf generally possess a fresher, sweeter, and smoother taste, while mature leaves tend to exhibit a more intense herbal aroma, accompanied by increased astringency and a rougher taste [15]. This sensory difference is closely associated with underlying metabolic patterns, in which tender leaves are characterized by higher levels of free amino acids and terpene-derived volatile compounds, whereas mature leaves tend to accumulate higher levels of flavonol glycosides and lipid oxidation-related volatile components [16,17].

Although both cultivar and leaf maturity exert strong influences on white tea quality, existing studies have often examined these factors separately or have focused on a limited subset of chemical constituents. Consequently, the metabolic mechanisms linking cultivar-specific pathways with maturity-driven compositional shifts remain incompletely understood. In particular, studies that simultaneously integrate volatile and non-volatile profiling across multiple cultivars and maturity grades within a unified chemical–sensory framework remain limited. This limitation constrains a more comprehensive understanding of how these interacting biological factors jointly shape white tea flavor.

To address this gap, we conducted an integrated flavor investigation using twelve representative white tea samples encompassing four cultivars and three grades. Non-volatile metabolites were characterized using ultra-high-performance liquid chromatography–quadrupole exactive orbitrap mass spectrometry (UHPLC-Q Exactive-Orbitrap MS), and volatile compounds were profiled using headspace solid-phase microextraction coupled with gas chromatography-mass spectrometry (HS-SPME-GC-MS). Sensory attributes were quantified through descriptive analysis. By aligning these datasets with multivariate modeling, we aimed to systematically disentangle the respective and joint contributions of cultivar and leaf maturity to white tea flavor.

## 2. Materials and Methods

### 2.1. Sample Collection and Classification of White Tea

During April 2024, fresh tea raw materials were selectively harvested from four representative white tea cultivars in Fujian Province, namely FADB, ZHDB, FDDB, and FDDH. For each cultivar, fresh leaves of three maturity grades were collected: Grade 1 (single bud), Grade 2 (one bud with one to two leaves), and Grade 3 (one bud with three to four leaves), yielding a total of 12 white tea samples (4 cultivars × 3 grades). Details of the tea samples are provided in Table S1. All raw materials were processed using the local standardized white tea manufacturing procedures practiced in Fujian, including withering and drying, to obtain the final dry tea samples used for subsequent analyses (Figure S1).

### 2.2. Chemicals and Reagents

Certified reference standards were employed for the identification and quantification of major non-volatile constituents in white tea, including catechins, theaflavins, amino acids, and other phenolic compounds. These standards supported targeted UHPLC–MS analysis as well as colorimetric and physicochemical determinations. LC–MS-grade organic solvents were used for non-volatile metabolomic analysis, and GC–MS-grade reagents and n-alkane standards were applied for volatile compound identification and retention index calculation. Analytical-grade reagents were used for routine physicochemical assays, including total phenolics, total flavonoids, amino acids, protein-related measurements, and mineral element analysis. Detailed information regarding specific reference compounds, solvents, reagents, and their suppliers is provided in Section S1 (Supplementary Materials).

### 2.3. Quantitative Determination of Physicochemical Parameters

Total polyphenol, protein, amino acid nitrogen, free amino acids, moisture content, theanine, theaflavins, total flavonoids, and mineral elements were measured according to the Chinese national or industry standard methods (Table S2). Catechins and caffeine, including catechin (C), epicatechin (EC), epigallocatechin (EGC), epicatechin gallate (ECG), epigallocatechin gallate (EGCG), gallocatechin gallate (GCG), gallic acid (GA), and caffeine, were quantified using a validated in-house ultra-performance liquid chromatography-photodiode array (UPLC-PDA) method. Chromatographic separation was achieved on a Waters ACQUITY UPLC system equipped with an ACQUITY UPLC HSS T3 column (1.8 μm, 2.1 mm × 100 mm; Waters, Milford, MA, USA). The mobile phases comprised solvent A (9% acetonitrile, 2% formic acid, and 20 μg/mL EDTA in water, *v*/*v*) and solvent B (80% acetonitrile, 2% formic acid, and 20 μg/mL EDTA in water, *v*/*v*). The flow rate was maintained at 0.4 mL/min, with an injection volume of 2.5 μL. The gradient elution program for solvent B was as follows: 0% (0–2.5 min), 15% (2.5–5.0 min), 72% (5.0–6.0 min), and 0% (6.0–8.0 min), succeeded by column re-equilibration at 0% B. Detection was performed with a photodiode-array detector, and quantification was accomplished by external calibration with mixed reference standards.

### 2.4. Sensory Quality Evaluation and Quantitative Descriptive Analysis (QDA)

Tea infusions were prepared by steeping 3.0 g of dried tea leaves in 150 mL of boiling water for 5 min at room temperature, following GB/T 23776-2003 [18]. Sensory evaluation was conducted by a trained expert panel using Quantitative Descriptive Analysis (QDA) [19]. Before formal assessment, panelists underwent standardized training to develop a consensus vocabulary and to calibrate the perception of key white-tea aroma and taste attributes. During evaluation, each descriptor was quantitatively rated on a 0–5 intensity scale, and mean panel scores were used for statistical analyses. All participants took part voluntarily and provided informed consent before the evaluation.

### 2.5. UHPLC-Q Exactive-Orbitrap MS-Based Untargeted Metabolomic Analysis

White tea leaves were ground into a fine powder, and 100 mg of the powder was extracted with 4 mL of 70% methanol using ultrasonic-assisted extraction at room temperature for 40 min, following the procedure of [20] with minor modifications. After centrifugation at 12,000 rpm, the clarified supernatant was collected and used for untargeted metabolomic analysis. This extraction protocol ensured efficient recovery of non-volatile secondary metabolites while minimizing thermal degradation.

Untargeted metabolomic profiling of non-volatile compounds in tea infusions was performed using a UHPLC-Q Exactive-Orbitrap MS (Thermo Fisher Scientific, Rockford, IL, USA), following a previously described method with minor modifications [21]. Chromatographic separation was conducted on an ACQUITY UPLC HSS T3 column maintained at 40 °C, with the autosampler temperature set to 10 °C. The mobile phase consisted of 0.1% formic acid in water (A) and acetonitrile (B). The flow rate was 0.3 mL/min, and the injection volume was 3 µL. The gradient program for solvent B was as follows: 5% (0–0.5 min), 10% (1.5 min), 30% (5.0 min), 40% (7.0 min), 60% (9.0 min), 85% (10.5 min), and 95% (11.0–12.5 min), followed by re-equilibration at 5% (13.5–15.0 min).

Mass spectrometric detection was carried out using an electrospray ionization (ESI) source operating in negative-ion mode, with a spray voltage of 3.2 kV. The resolutions for full MS and data-dependent MS/MS (ddMS^2^) were set to 70,000 and 35,000 (at *m*/*z* 200), respectively. The capillary temperature was 320 °C, and the auxiliary gas-heater temperature was 300 °C. Sheath and auxiliary gas flow rates were 40 and 10 arbitrary units, respectively. The scan range was *m*/*z* 100–1500.

Raw UHPLC-MS data were processed using Compound Discoverer™ 3.2.0 (Thermo Fisher Scientific). The data-processing workflow included peak detection, deconvolution, retention-time alignment, background subtraction, and preliminary annotation using mzCloud and ChemSpider. The aligned feature table generated by Compound Discoverer served as the primary dataset for untargeted metabolomic analysis and differential screening of metabolites. Metabolite identification was based on Compound Discoverer annotations, integrating accurate mass, isotopic pattern, and MS/MS spectral matching to support putative metabolite assignments.

### 2.6. Volatile Metabolomic Profiling by HS-SPME-GC-MS

Volatile profiling was conducted using HS-SPME-GC-MS with modifications based on the previously established method [22]. Briefly, 0.5 g of tea powder was placed into a 20 mL headspace vial, followed by the addition of 5 mL of hot water. Cyclohexanone was spiked into each vial as the internal standard (0.3788 μg/mL, 20 μL). The vial was immediately sealed and equilibrated at 45 °C for 15 min, after which the SPME fiber was exposed to the headspace for 40 min for extraction.

GC-MS analysis was performed on a TRACE 1300 gas chromatograph coupled with an ISQ single-quadrupole mass spectrometer (Thermo Fisher Scientific) equipped with a HP-INNOWAX capillary column (30 m × 0.25 mm i.d. × 0.25 μm film). The SPME fiber was thermally desorbed in the injector at 250 °C for 5 min. Helium was used as the carrier gas at a constant flow rate of 1.0 mL/min. The oven temperature program was: 45 °C for 8 min; ramped to 160 °C at 5 °C/min and held for 20 min; then ramped to 250 °C at 12 °C/min and held for 5 min. The MS was operated in electron ionization (EI, 70 eV) mode with full-scan acquisition over *m*/*z* 42–500. The transfer line and ion source temperatures were set to 280 °C and 230 °C, respectively.

Chromatographic data were processed using TraceFinder™ 5.0 software (Thermo Fisher Scientific). Volatile compounds were identified by matching the obtained mass spectra with the NIST 17 library database. Additionally, linear retention indices were calculated using a homologous series of C_7_–C_40_ *n*-alkanes run under the same GC conditions.

Quantification of volatile organic compounds (VOCs) was achieved using the internal standard method. The relative concentration (*C*_*i*_) of each volatile compound was calculated according to Equation (1):

(1) $$C_{i}=\frac{A_{\mathrm{unknown}}}{A_{\mathrm{IS}}}\times C_{\mathrm{IS}}$$

where $A_{\mathrm{unknown}}$ and $A_{\mathrm{IS}}$ are the GC peak areas of the target compound and the internal standard (cyclohexanone), respectively, and $C_{\mathrm{IS}}$ is the known concentration of the internal standard.

The contribution of each volatile to the overall aroma was evaluated by its odor activity value (OAV), which was calculated according to Equation (2):

(2) $${\mathrm{OAV}}_{i}=\frac{C_{i}}{OT_{i}}$$

where $C_{i}$ is the concentration of compound *i* (in μg/L) and ${OT}_{i}$ is the odor threshold of that compound in water. Compounds with OAV > 1 were considered to be odor-active constituents that significantly influence the tea’s aroma profile.

### 2.7. Statistical Analysis

Raw chromatographic and mass spectrometric data acquired from GC-MS and UHPLC-MS were initially processed using Xcalibur (Thermo Fisher Scientific) to generate peak tables. These peak tables were subsequently imported into SIMCA 14.1 (Umetrics, Umeå, Sweden) for multivariate statistical analyses. Principal component analysis (PCA) was first employed to provide an overview of the data structure, to facilitate the identification of clustering patterns, and to detect potential outliers. Following this exploratory analysis, partial least squares discriminant analysis (PLS-DA) was conducted to maximize group discrimination and to elucidate the variables contributing to sample separation. For the selection of discriminant markers, metabolites were filtered based on variable importance in projection (VIP) values derived from the respective PLS-DA models. Specifically, volatile metabolites were retained with VIP values ≥ 1.0, while a more stringent threshold of VIP ≥ 1.5 was applied for non-volatile metabolites.

For in-depth exploration of component and correlation patterns, intensity heatmaps and correlation heatmaps were constructed using the pheatmap and corrplot packages in R (version 4.3.0), respectively. Before heatmap generation, all physicochemical parameters and metabolite abundances (including both volatile and non-volatile metabolites) were standardized by Z-score normalization to facilitate unbiased comparisons across variables. Finally, univariate statistical analyses were performed using GraphPad Prism 8 (GraphPad Software, San Diego, CA, USA) to assess the statistical significance of observed differences among groups.

## 3. Results

### 3.1. Integrated Quality Characteristics of White Tea Samples Across Grades and Cultivars

**Figure 1:**
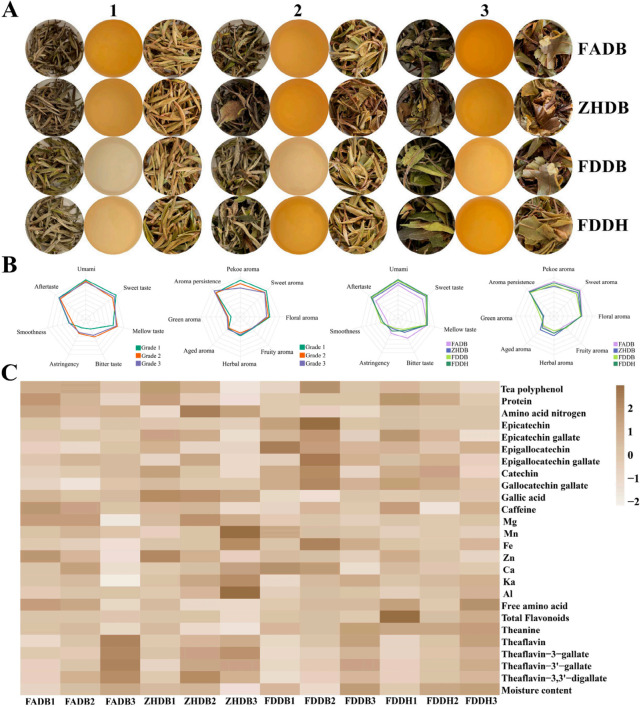
Appearance, sensory characteristics, and physicochemical attributes of white teas. (**A**) Representative dry-leaf appearance and infusion color of four cultivars across three grades. Numbers 1–3 indicate increasing leaf maturity levels, corresponding to Grades 1–3. (**B**) Radar charts of sensory profiles summarized by grade and cultivar, illustrating key taste and aroma attributes. (**C**) Heatmap of physicochemical parameters (*Z*-score standardized), including catechins, caffeine, tea polyphenols, amino acids, theaflavins, mineral elements, and moisture content.

Figure 1A illustrates that the twelve white tea samples exhibited clear differences in liquor color. FADB and ZHDB produced from orange-yellow to dark-yellow infusions, whereas FDDB and FDDH yielded paler yellow to apricot-yellow liquors. Within each cultivar, Grade 1 consistently had the lightest color, and Grades 2 and 3 showed progressively deeper hues. These patterns indicated that cultivar set the baseline pigmentation, while leaf maturity contributed to the stepwise increase in color intensity across grades. The gradual deepening of liquor color with grade was consistent with biochemical changes reported during leaf maturation, including intensified phenolic oxidation and increased formation of oxidized flavonoid pigments [14,23].

Sensory evaluations revealed distinct and cultivar-specific flavor signatures (Figure 1B). FADB displayed pronounced bitterness and astringency, FDDB exhibited strong sweetness and umami, FDDH maintained a more balanced sensory profile, and ZHDB showed characteristic aged, woody, and medicinal aromas. These sensory traits were in line with known differences in phenolic load, nitrogenous compounds, and volatile composition among tea germplasms [12]. Across grades, the flavor trajectory followed a consistent direction: Grade 1 teas had higher sweetness, umami, and aftertaste persistence, whereas Grades 2 and 3 exhibited increased bitterness and astringency, as well as reduced sweetness. Aroma also shifted from delicate floral-fruit notes in Grade 1 to herbal or slightly aged notes in Grade 3. This shift changes in terpene biosynthesis, fatty-acid-derived volatiles, and glycosidically bound precursors as leaves mature [24].

Physicochemical measurements were consistent with the sensory patterns across grades (Figure 1C). Grade 1 teas contained higher levels of free amino acids, theanine, and soluble proteins, which contribute directly to sweetness and umami. These nitrogenous constituents declined progressively with maturity. Catechin contents differed among cultivars but followed a uniform downward trend from Grade 1 to Grade 3, while oxidized phenolics increased. This metabolic shift agrees with evidence that catechin biosynthesis decreases and oxidative degradation intensifies during leaf maturation [14,23]. The joint decrease in amino acids and rise in oxidized phenolics provides a biochemical basis for the transition from sweet-umami profiles in Grade 1 to more astringent and green attributes in lower grades.

Cultivar differences were evident in addition to these maturity-driven trends. FDDB and FDDH retained higher catechin levels, whereas FADB and ZHDB exhibited lower monomeric catechins and stronger oxidative signatures. ZHDB also showed marked enrichment of Mn, Fe, and Zn, consistent with the mineral-rich soils in Zhenghe County [25]. Since Mn and Fe are cofactors of polyphenol oxidase (PPO) and polyphenol oxidase (POD), their higher abundance may enhance oxidative capacity during withering [23]. This mineral-associated increase in redox potential helps explain the deeper color and more pronounced oxidative notes characteristic of ZHDB, especially in mature leaves where phenolics are more vulnerable to enzymatic conversion.

Collectively, these quality data confirm a two-level organization of white tea characteristics: cultivar sets the sensory framework through inherent phenolic, mineral, and aromatic biosynthetic capacities, and leaf maturity determines the direction and magnitude of biochemical shifts within this framework. This layered structure offers a mechanistic foundation for interpreting the metabolomic patterns presented in subsequent sections.

### 3.2. Overview of Metabolomic Variation Across Cultivars and Grades

**Figure 2:**
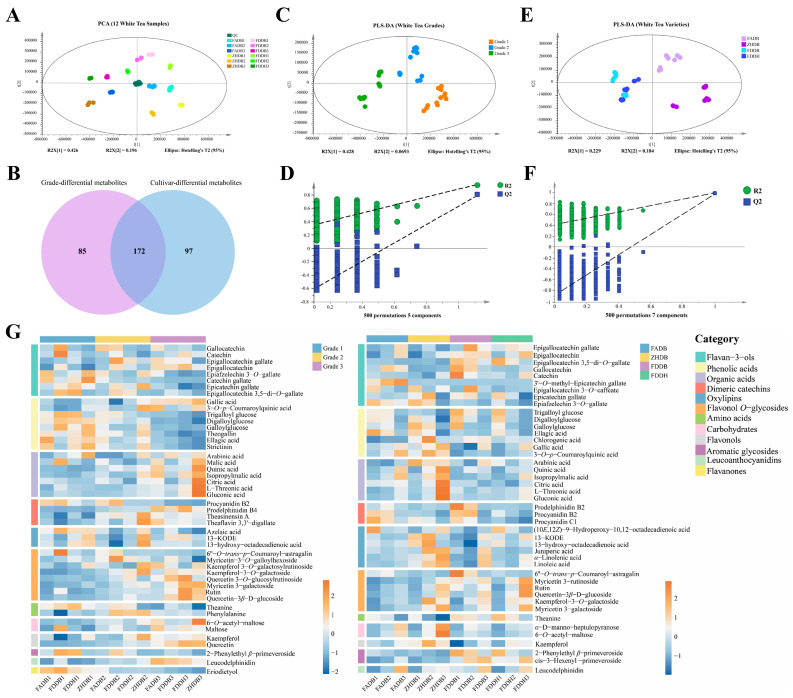
Multivariate statistical analysis of non-volatile metabolites in white tea samples. (**A**) PCA score plot of 12 white tea samples; the ellipse indicates the 95% Hotelling’s T^2^ confidence region, and quality control (QC) samples cluster near the origin. (**B**) Venn diagram showing the overlap between grade-related and cultivar-related metabolites. (**C**) PLS-DA score plot discriminating white tea grades (Grades 1–3). (**D**) Permutation test (500 permutations) for the grade PLS-DA model. The dashed lines represent the regression lines of R^2^ and Q^2^ values obtained from permutation testing. (**E**) PLS-DA score plot discriminating cultivars (FADB, ZHDB, FDDB, and FDDH). (**F**) Permutation test (500 permutations) for the cultivar PLS-DA model. The dashed lines represent the regression lines of R^2^ and Q^2^ values obtained from permutation testing. (**G**) Heatmaps of differential non-volatile metabolites in white teas across different grades and cultivars (VIP ≥ 1.5, *p* < 0.05).

The PCA model provided an overall view of metabolic variation among the twelve samples (Figure 2A). The samples did not form completely separated clusters, but grade differences produced broader dispersion across the PCA space than cultivar differences. Cultivar-related shifts occurred within each grade rather than across the main axes. This pattern indicates that leaf maturity contributes more strongly to global metabolic variance than genetic background. QC samples clustered tightly, confirming the reliability of the analytical workflow. The dominance of maturity in shaping the PCA structure agrees with known physiological patterns during leaf development. Earlier studies have reported that nitrogen assimilation and amino acid biosynthesis generally decline as leaves mature, whereas phenolic oxidation and carbohydrate turnover increase [26]. This pattern reflects a developmental shift from growth-supporting primary metabolism toward secondary metabolic processes associated with structural reinforcement and defense. Our PCA map reflects these biochemical tendencies: Grade 1 samples cluster closer to nitrogen-rich metabolic profiles, while Grade 3 samples shift toward profiles enriched in oxidized phenolics and organic acids. These relationships align with reports that catechin biosynthesis decreases and oxidative degradation intensifies with increasing leaf age [14,23].

The Venn diagram (Figure 2B) summarizes the metabolites selected by SIMCA with VIP values of at least 1.5. Under this criterion, 257 metabolites were associated with grade and 269 with cultivar, and 172 metabolites were shared by both groups. This result shows that many metabolites are influenced by both factors. In other words, grade and cultivar do not act on completely separate metabolite sets, but rather affect several common pathways to different degrees.

PLS-DA further clarified the contributions of each factor (Figure 2C–F). The grade-based model achieved complete separation of Grade 1, Grade 2, and Grade 3, demonstrating that maturity triggers a system-level reorganization of the non-volatile metabolome. The cultivar-based model also showed separation, but the clusters were closer and partially overlapping. This supports the idea that cultivar mainly affects specific branches of metabolic pathways, such as methylation, glycosylation, organic acid metabolism, and lipid oxidation, while maturity governs broader shifts in nitrogen metabolism, catechin turnover, and phenolic oxidation. These relationships are consistent with developmental and genetic studies in tea that describe maturity as the primary metabolic driver and cultivar as a modifier of pathway-specific outputs [14,23,26]. Overall, the PCA and PLS-DA results revealed a hierarchical structure in white tea metabolomics. Leaf maturity determines the major biochemical trajectory, while cultivar fine-tunes specific metabolite groups within this framework.

### 3.3. Grade- and Cultivar-Associated Non-Volatile Metabolites

#### 3.3.1. Grade-Related Metabolites

A total of 47 metabolites were associated with grade differences, which can be fundamentally interpreted as maturity-driven metabolic variation reflecting differences in leaf developmental stage. These metabolites spanned twelve chemical classes, including flavan-3-ols, phenolic acids, organic acids, flavonol *O*-glycosides, catechin dimers, oxylipins, amino acids, carbohydrates, flavonols, aromatic glycosides, leucoanthocyanidins, and flavanones (Table S3). Their abundance profiles showed a highly ordered, maturity-related trajectory (left panel of Figure 2G). Galloylated catechins displayed distinct grade-specific patterns: EGCG was most abundant in Grades 2–3, ECG in Grades 1–2, and EGC in Grade 3, while epigallocatechin-3,5-di-*O*-gallate was strongly enriched in Grade 1. Phenolic acids exhibited a similar developmental ordering, with theogallin, digalloylglucose, and trigalloylglucose predominant in Grade 1, and free gallic acid increasingly enriched in Grade 3. Organic acids, particularly quinic acid, also showed strong maturity dependence and reached their highest levels in Grade 3, most notably in ZHDB3. Catechin dimers displayed intermediate behaviors: procyanidin B2 was enriched in Grades 1–2, whereas theasinensin A peaked in Grade 2.

In contrast, mature-leaf metabolites such as kaempferol- and quercetin-derived *O*-glycosides accumulated predominantly in Grade 3, consistent with the established upregulation of flavonol biosynthesis and uridine diphosphate–dependent glycosyltransferase (UGT) activity during leaf maturation [27]. Collectively, these distributions indicate a coordinated transition in which young-leaf metabolites, including free amino acids, monomeric catechins, galloylglucose esters, and several catechin dimers, progressively decline from Grade 1 to Grade 3, whereas oxidized phenolics, flavonol *O*-glycosides, gallic acid, and organic acids increase.

These metabolite trends reflect known developmental mechanisms. Young leaves exhibit high nitrogen assimilation capacity and enhanced biosynthesis of amino acids such as theanine, supported by strong activity of glutamine synthetase and serine hydroxymethyltransferase [26]. As leaves mature, nitrogen mobilization and amino acid formation decline, reducing sweetness, umami, and the buffering capacity against phenolic astringency. Parallel shifts occur in the flavonoid pathway. Catechin biosynthesis decreases markedly with leaf expansion due to reduced expression of chalcone synthase (CHS), anthocyanidin reductase (ANR), and leucoanthocyanidin reductase (LAR), while oxidative and hydrolytic processes intensify [14,23]. Accelerated de-galloylation and oxidation in mature tissues explain the observed decrease in galloylated catechins and the rise in gallic acid and flavonol glycosides.

These changes provide a mechanistic explanation for the sensory differences among grades. High levels of amino acids and galloylated catechins in Grade 1 contribute to sweetness, umami, and balanced astringency. Grade 3 samples contain fewer amino acids and higher amounts of flavonol *O*-glycosides and oxidized phenolics, which generate more persistent and less buffered astringency. This interpretation aligns with oral-metabolomics evidence showing that galloylated catechins are strongly modulated by amino acids, whereas flavonol *O*-glycosides induce sustained oral friction with limited biochemical buffering [28].

#### 3.3.2. Cultivar-Related Metabolites

Table S4 summarizes the 44 cultivar-associated metabolites, mainly including eight flavan-3-ols, seven phenolic acids, six organic acids, six oxylipins, six flavonol *O*-glycosides, and three catechin dimers, together with smaller numbers of carbohydrates, flavonols, aromatic glycosides, and leucoanthocyanidins. The abundance patterns of these metabolites (right panel of Figure 2G) showed a structurally diverse organization that reflected genotype-related regulation rather than the ordered maturity-driven changes described for grade.

Among flavan-3-ols, epigallocatechin-3,5-di-*O*-gallate, EGC, and EGCG had the highest VIP values and were predominantly enriched in FDDB and FDDH. Within these cultivars, epigallocatechin-3,5-di-*O*-gallate decreased from Grade 1 to Grade 3, EGC increased with grade, and EGCG was most abundant in Grade 2. In contrast, epiafzelechin 3-*O*-gallate and 3′-*O*-methyl-Epicatechin gallate were characteristic of FADB. Epiafzelechin 3-*O*-gallate declined with grade, whereas 3′-*O*-methyl-Epicatechin gallate increased. ECG was mainly present in Grades 1–2 of ZHDB, FDDB, and FDDH. Catechin dimers further differentiated the cultivars: procyanidin B2 was enriched in Grades 1–2 of FADB and FDDB, while prodelphinidin B2 was concentrated in Grades 1–2 of FDDB. These patterns indicate that each cultivar allocates flavan-3-ol flux differently among monomers, galloylated derivatives, and dimers, and that internal grade trends vary in both direction and magnitude across cultivars.

Organic and phenolic acids also showed clear cultivar specificity. Quinic acid and citric acid accumulated strongly in ZHDB, especially in ZHDB3, while trigalloylglucose was most abundant in Grade 1 of FDDB and FDDH, and gallic acid consistently reached its highest levels in ZHDB3. This combination of enhanced organic acid pools and advanced phenolic hydrolysis indicates that ZHDB leaves undergo more extensive oxidative and hydrolytic transformation, which is consistent with their deeper liquor color and more pronounced aged or woody notes.

Oxylipins provided an additional layer of separation. ZHDB, particularly ZHDB2, accumulated high levels of 13-KODE and 13-hydroxy-octadecadienoic acid. These metabolites are products of linoleic acid oxidation through the lipoxygenase pathway, and their enrichment supports reports that lipid oxidation efficiency differs among tea cultivars [29]. The strong oxylipin signature in ZHDB suggests that this cultivar has a greater tendency to channel fatty-acid-derived flux into oxidative lipid products, which aligns with its greener and more resinous aroma style.

Flavonol *O*-glycosides, including myricetin-3-galactoside, kaempferol-3-*O*-galactoside, rutin, and quercetin-3-*O*-glucoside, also contributed to varietal separation. Kaempferol derivatives were particularly prominent in ZHDB, pointing to cultivar-specific enhancement of flavonol biosynthesis and glycosylation. At the same time, FDDB and FDDH showed strong accumulation of aromatic glycosides, including 2-phenylethyl *β*-primeveroside and cis-3-hexenyl primeveroside, which indicates a higher capacity for storing glycoside-bound aroma precursors. This pattern is consistent with studies showing that glycosyltransferase activity and glycoside storage vary across tea germplasm [7,19] and helps explain the sweeter and more floral aroma profiles of FDDB and FDDH.

Viewed at the cultivar level, these metabolite distributions form coherent chemical profiles. FADB is characterized by methylated and caffeoylated catechin derivatives, in line with reports that *O*-methyltransferase and acyltransferase gene expression differ among tea cultivars [30]. ZHDB is distinguished by its combination of organic acids, oxylipins, carbohydrates, and advanced phenolic acids, reflecting a stronger oxidative and hydrolytic background. FDDB stands out through its high levels of catechins, catechin dimers, and aroma glycosides, whereas FDDH combines theanine, epigallocatechin-3,5-di-*O*-gallate, flavonoid glycosides, and aroma glycosides, consistent with a softer taste and floral-sweet aromatic style.

These patterns indicate that cultivar differences do not arise from uniform increases or decreases across the metabolome. Instead, each genetic background selectively enriches particular metabolic branches, such as methylation, acylation, lipid oxidation, or glycosylation. These metabolic branches provide the biochemical basis for the cultivar-specific flavor and aroma profiles.

### 3.4. Volatile Composition and Odor Activity of White Tea

**Figure 3:**
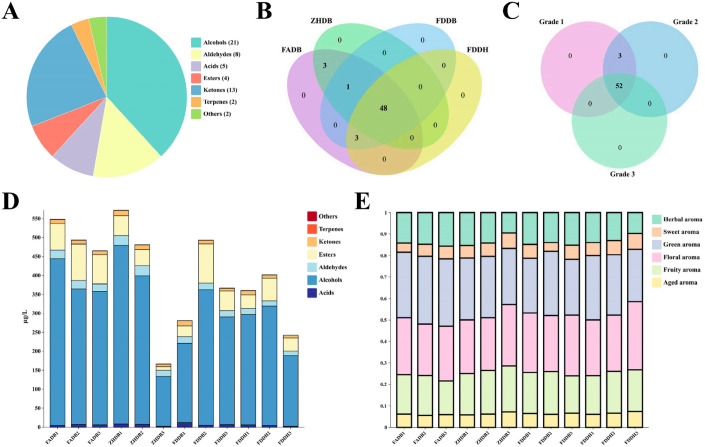
Characterization of volatile metabolite composition and aroma attributes in Fujian white teas. (**A**) Proportional distribution of major volatile chemical classes. Numbers in parentheses indicate the number of metabolites identified in each class. (**B**) Venn diagram of cultivar-related volatile metabolites detected in four white tea cultivars (FADB, ZHDB, FDDB, and FDDH). Colored circles represent different cultivars, and numbers indicate the count of shared or unique metabolites. (**C**) Venn diagram of grade-related volatile metabolites across three grades (Grades 1–3). Colored circles represent different grades, and numbers indicate the count of shared or unique metabolites. (**D**) Stacked bar plots of the relative contents of volatile chemical classes in each sample. Terpenes (orange-red) and Others (red) occur at trace levels and are not visually distinguishable in the stacked bars. (**E**) Relative contributions of six aroma note categories across the twelve white tea samples.

A total of 55 volatile compounds were identified and classified into seven chemical categories according to Zhai’s criteria [31], comprising 21 alcohols, 8 aldehydes, 5 acids, 4 ketones, 4 esters, 2 terpenes, and 2 compounds assigned to “others” (Figure 3A and Table S5). Alcohols were the dominant class, followed by aldehydes, confirming that these two groups constitute the core aromatic backbone of white tea. The Venn diagrams (Figure 3B,C) show that 48 volatiles were shared by all cultivars and 52 were shared across grades, indicating that white tea aroma is built on a conserved volatile skeleton. Differences between cultivars and grades arise mainly from quantitative shifts rather than the presence or absence of specific compounds.

Semi-quantitative data illustrated distinct grade-related tendencies (Figure 3D,E). Grade 1 samples contained higher levels of floral-fruity monoterpenoids such as linalool, geraniol, and (*Z*)-linalool oxide. These compounds are products of terpene synthase (TPS)-mediated biosynthesis and hydrolysis of glycosidically bound precursors, both of which are more active in young leaves where precursor pools are larger [32,33]. This molecular pattern corresponds well to the sweet-floral aroma perceived in Grade 1.

Grade 3 showed higher proportions of aldehydes and ketones, including hexanal, octanal, and 2-heptanone. These are characteristic products of lipoxygenase (LOX)-mediated lipid oxidation, which increases during leaf maturation and senescence [34]. Their accumulation explains the greener, slightly fatty or woody notes of lower grades. These findings support a maturity-driven aromatic progression: young leaves favor TPS-derived compounds, whereas mature leaves favor LOX-derived volatiles.

OAV analysis identified only a small number of volatiles with strong sensory impact (Table S6). Linalool showed very high OAVs across all cultivars, underscoring its central role in shaping the floral identity of white tea. (*E*,*Z*)-3,6-Nonadien-1-ol exhibited exceptionally high potency due to its extremely low odor threshold and was a key contributor to the green notes of ZHDB. Geraniol, 6-methyl-5-hepten-2-one, and 2-octanone also contributed measurably to citrus-floral or fatty nuances, while many commonly detected volatiles such as 1-nonanol, (*Z*)-jasmone, and *p*-cymene had OAVs near or below 1 and therefore contributed little to overall aroma.

Clear cultivar specificity emerged from the combined profiling and OAV analysis. FDDB and FDDH showed strong contributions from floral alcohols and glycoside-derived monoterpenoids, consistent with their sweet-floral sensory profiles [35]. FADB exhibited stronger oxidized or citrus-tinged notes due to higher levels of linalool oxides and related monoterpenoid derivatives. ZHDB accumulated the highest amounts of LOX-derived aldehydes and (*E*,*Z*)-3,6-nonadien-1-ol, giving a greener and more resinous aroma. These patterns align with the cultivar-related differences in glycoside storage, monoterpene metabolism, and lipid oxidation reported for tea germplasm [36].

To sum up, the volatile results demonstrate that the aromatic identity of white tea is governed by a conserved core volatile set. Grade modulates the relative balance between TPS- and LOX-derived pathways, while cultivar determines which branches of this volatile network are emphasized through differences in precursor pools, glycosylation capacity, and lipid oxidation efficiency. This framework highlights a small group of high-impact volatiles that play disproportionate roles in defining cultivar-specific aroma styles and therefore represent practical targets for breeding and processing optimization.

### 3.5. Multivariate Analysis of Volatile Profiles in Relation to Grade and Cultivar

**Figure 4:**
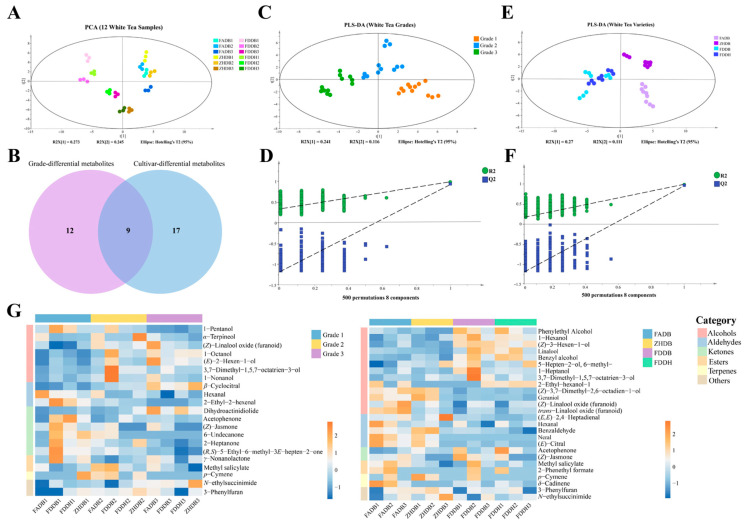
Multivariate statistical analysis of volatile metabolites in white tea samples. (**A**) PCA score plot of 12 white tea samples based on volatile metabolite profiles; the ellipse indicates 95% Hotelling’s T^2^ confidence region. (**B**) Venn diagram showing the overlap between grade-differential and cultivar-differential volatile metabolites. (**C**) PLS-DA score plot discriminating white tea grades (Grades 1–3). (**D**) Permutation test (500 permutations) for the grade PLS-DA model. The dashed lines represent the regression lines of R^2^ and Q^2^ values obtained from permutation testing. (**E**) PLS-DA score plot discriminating cultivars (FADB, ZHDB, FDDB, and FDDH). (**F**) Permutation test (500 permutations) for the cultivar PLS-DA model. The dashed lines represent the regression lines of R^2^ and Q^2^ values obtained from permutation testing. (**G**) Heatmaps of differential volatile metabolites in white teas across different grades and cultivars (VIP ≥ 1.0, *p* < 0.05).

Multivariate modeling of the volatile data provided a complementary perspective on how aroma differentiation emerges among grades and cultivars. PCA revealed that Grade 1 samples clustered closely in the score space (Figure 4A), reflecting a highly consistent aromatic profile across cultivars. This consistency is aligned with the chemical characteristics of young shoots, which typically possess high pools of terpene alcohols and early-stage monoterpene oxidation products such as linalool, (*Z*)-linalool oxide, 1-pentanol, and hexanal [37]. Their uniform distribution suggests that early volatile metabolism is strongly developmentally constrained. In contrast, Grades 2 and 3 formed progressively broader clusters, indicating that as leaves mature, cultivar-related metabolic variation becomes more pronounced. The increased dispersion coincides with rising levels of aldehydes, ketones, and LOX-mediated oxidation products, which are known to be more sensitive to genotype as metabolic diversity expands with leaf age.

The Venn diagram (Figure 4B) shows that most VIP-selected discriminatory volatiles were shared between grade and cultivar, whereas only a limited number were specific to a single factor. This pattern indicates that grade and cultivar primarily drive quantitative variation in volatile composition within a largely conserved framework, rather than inducing qualitative differences in volatile identity.

The grade-based PLS-DA model produced complete separation among Grades 1, 2, and 3 (Figure 4C), and permutation validation confirmed its robustness (Figure 4D). Grade 1 teas consistently displayed high levels of floral-fresh compounds, including linalool, 1-hexanol, 1-heptanol, and a group of C6 aldehydes commonly associated with green-fresh notes. Grade 2 teas were characterized by increased amounts of floral-fruity alcohols and esters such as 3,7-dimethyl-1,5,7-octatrien-3-ol, 1-nonanol, 1-octanol, methyl salicylate, and *p*-cymene, consistent with an intermediate aromatic stage between the freshness of Grade 1 and the oxidative sharpness seen in Grade 3. Grade 3 samples contained the most diverse set of oxidative volatiles, including (*Z*)-linalool oxide (furanoid), (*E*)-2-hexen-1-ol, *β*-cyclocitral, and dihydroactinidiolide. These compounds correspond to grassy, resinous, or slightly aged notes characteristic of mature leaves. ZHDB3 additionally showed elevated *N*-ethylsuccinimide, which distinguished it from other Grade 3 samples.

The cultivar-based PLS-DA model also produced valid separation, revealing stable and internally coherent aromatic configurations for each cultivar (Figure 4E,F). FADB was characterized by higher concentrations of oxidized monoterpenes and norisoprenoids, including (*Z*)- and trans-linalool oxide, neral, *E*-citral, and related derivatives, giving rise to a floral-citrus profile with subtle aged nuances. ZHDB displayed strong enrichment in LOX-derived aldehydes such as hexanal and octanal, as well as benzaldehyde, *p*-cymene, and the potent green volatile (*E*,*Z*)-3,6-nonadien-1-ol. These features are consistent with the cultivar’s grassy-resinous sensory attributes. FDDB showed pronounced accumulation of floral and benzenoid alcohols, including linalool, benzyl alcohol, 1-heptanol, 1-hexanol, and methyl salicylate, generating a softer floral-sweet aroma. FDDH exhibited a similar but slightly milder pattern, which corresponded to their proximity in the multivariate space.

When these results are considered together, a hierarchical structure becomes evident. At the physiological level, leaf maturity establishes the dominant aromatic trajectory by shifting the balance from terpene alcohols and early oxidation products in young leaves to aldehydes, ketones, and norisoprenoids in mature leaves. Cultivar-specific regulation then modulates this developmental trajectory by emphasizing different biosynthetic branches, including monoterpene oxidation, lipid-derived aldehyde formation, and glycoside accumulation. This mechanism explains why Grade 1 samples across cultivars express a highly similar aromatic backbone, why Grades 2 and 3 diverge more strongly, and why cultivars maintain distinctive aromatic identities even when harvested at the same developmental stage. These observations are consistent with established biochemical models in tea, which describe a decline in terpene synthase activity and glycosidically bound precursors with increasing leaf age, accompanied by enhanced LOX-mediated lipid oxidation. Together, these findings outline a coherent framework for understanding how developmental and genetic factors jointly determine the volatile profiles and sensory styles of white tea.

### 3.6. Cross-Layer Correlations Between Non-Volatile and Volatile Metabolites

**Figure 5:**
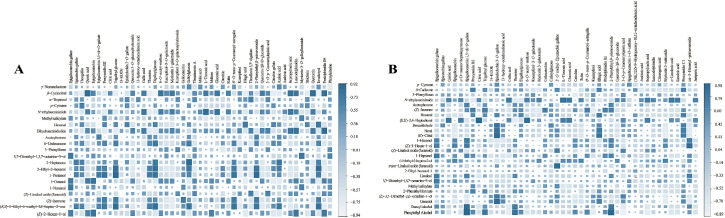
Correlation heatmaps between differential non-volatile and volatile metabolites in white tea. (**A**) Pearson correlations between grade-related differential non-volatile metabolites and grade-related differential volatile compounds. (**B**) Pearson correlations between cultivar-related differential non-volatile metabolites and cultivar-related differential volatile compounds.

Cross-layer correlation analysis revealed that the formation of white tea aroma is governed not by isolated precursor-product relationships, but by highly coordinated biochemical states shaped by leaf maturity and genetic background (Figure 5). Rather than forming diffuse or stochastic associations, volatile and non-volatile metabolites converged into a small number of biologically interpretable covariance structures, each representing a coherent metabolic condition of the leaf.

In the grade-oriented matrix (Figure 5A), a clear polarity emerged between a tender-leaf metabolic state and a mature-leaf oxidative state. EGCG, together with other young-leaf flavanols, showed strong positive correlations with floral-green volatiles such as 1-octanol, 1-nonanol, (*E*)-2-hexen-1-ol, and 3,7-dimethyl-1,5,7-octatrien-3-ol. This association reflects the biochemical environment of juvenile tissues, where catechin biosynthesis, glycosidically bound terpene hydrolysis, and early LOX turnover occur simultaneously. Conversely, gallic acid, quinic acid, gluconic acid, L-threonic acid, and multiple flavonol *O*-glycosides correlated strongly with *β*-cyclocitral and *N*-ethylsuccinimide, indicating that mature leaves undergo intensified phenolic hydrolysis and oxidative lipid fragmentation. Galloylated catechin esters (theogallin, digalloylglucose, trigalloylglucose) displayed intermediate correlations with C6/C9 aldehydes and selected terpenoid fragments, consistent with their biochemical role at the intersection of ester hydrolysis and oxidative branching. Collectively, these associations show that grade differentiation reflects a shift between two metabolically coherent states, rather than simple increases or decreases in individual compounds.

Cultivar-level correlations displayed a different organization, reflecting lineage-specific metabolic wiring (Figure 5B). EGCG again appeared as a central hub, but the volatiles with which it correlated varied by genetic background, supporting the view that cultivars differ in how flavanol metabolism is coupled to LOX and monoterpenoid pathways. Benzyl alcohol and phenylethyl alcohol showed strong covariance with procyanidin B2, epiafzelechin-3-*O*-gallate, and digalloylglucose, suggesting a cultivar-specific linkage between benzenoid formation and upstream flavanol intermediates. Organic acids, gallic acid, and carbohydrate derivatives clustered tightly with *N*-ethylsuccinimide and (*E*,*E*)-2,4-heptadienal, indicating that some cultivars coordinate oxidative volatile production with carbohydrate and phenolic turnover. A distinct set of correlations connected terpenoid aldehydes (neral, *E*-citral) to epiafzelechin-3-*O*-gallate and ellagic acid, revealing a cultivar-related alignment between phenylpropanoid and monoterpenoid metabolism.

Overall, the results indicate that non-volatile and volatile metabolites in white tea are not regulated in isolation, but instead assemble into coordinated and biologically coherent metabolic states. Grade differentiation is characterized by a shift from an early metabolic regime enriched in catechins and monoterpenes toward a later regime featuring enhanced hydrolytic and oxidative processes. Cultivar differentiation is manifested as lineage-specific differences in the coupling among phenolic turnover, lipid oxidation, and terpene biosynthesis. Together, these findings position leaf maturity and genetic background as two hierarchical but interacting axes of biochemical organization in white tea, while also defining the scope within which the present correlations can be interpreted.

The present study was conducted using representative white tea samples from major Fujian cultivars and grades, and the observed metabolic and sensory patterns should be interpreted within this defined material context. In addition, environmental and agronomic variables such as altitude, seasonal conditions, humidity, fertilization practices, and soil composition were not explicitly incorporated into the present experimental design and may act as additional sources of variation influencing tea chemical composition and sensory expression. While integrated metabolomic and sensory analyses revealed robust associations between chemical composition and flavor perception, direct experimental validation of specific pathway–sensory relationships was not addressed in the current work.

Future studies may extend these findings across broader geographic origins, processing practices, and harvest conditions to evaluate the generality of the identified metabolic states. Integrating targeted pathway validation, dynamic aroma release measurements, and consumer-oriented sensory assessment will further enable a more comprehensive understanding of how biochemical organization is translated into perceived flavor, thereby supporting the development of scientifically grounded strategies for quality evaluation and cultivar-oriented flavor design in white tea.

## 4. Conclusions

This study elucidated the relative roles of leaf maturity and cultivar background in shaping white tea flavor by integrating non-volatile and volatile metabolomics with sensory analysis. Flavor differentiation among grades was primarily structured by maturity-related changes in non-volatile composition, underpinning a systematic sensory transition from sweet and umami attributes in higher grades to more bitter and astringent sensations in lower grades. In parallel, grade-related variation in volatile composition was manifested as a shift from floral-fruit to more herbal aroma expression. Cultivar effects were less pronounced than maturity effects but were consistently expressed through pathway-level modulation, particularly within the volatile metabolome, contributing to stable varietal aroma characteristics within the same quality grade. Together, these findings provide a chemically grounded understanding of how grade and cultivar jointly shape taste and aroma characteristics in white tea, and offer a scientific reference for quality evaluation, raw material selection, and cultivar-oriented flavor management.

## Data Availability

The original contributions presented in this study are included in the article/Supplementary Material. Further inquiries can be directed to the corresponding author.

## Supplementary Materials

The following supporting information can be downloaded at: https://www.mdpi.com/article/10.3390/foods15030458/s1. Section S1: Detailed list of specific chemicals and reagents; Figure S1: Representative appearance of dry white tea samples classified by cultivar and grade; Table S1: Information and abbreviations of the twelve white tea samples categorized by cultivar and grade; Table S2: Physicochemical parameters and analytical methods used for white tea quality evaluation; Table S3: Key non-volatile metabolites (VIP ≥ 1.5) discriminating different grades of white tea; Table S4: Key non-volatile metabolites (VIP ≥ 1.5) discriminating different cultivars of white tea; Table S5: Volatile compounds with OAVs in samples exceeded 1; Table S6: Volatile compounds of white tea from different samples.

## Abbreviations

The following abbreviations are used in this manuscript:

AlCl_3_	Aluminum chloride
ANR	Anthocyanidin reductase
C	Catechin
CHS	Chalcone synthase
Ci	Concentration
ddMS^2^	Full MS and data-dependent MS/MS
EC	Epicatechin
ECG	Epicatechin gallate
EGC	Epigallocatechin
EGCG	Epigallocatechin gallate
EI	Electron ionization
ESI	Electrospray ionization
FADB	Fuan Dabai
FDDB	Fuding Dabai
FDDH	Fuding Dahao
GA	Gallic acid
GCG	Gallocatechin gallate
HPLC	High-performance liquid chromatography
HS-SPME-GC-MS	Headspace solid-phase microextraction coupled with gas chromatography-mass spectrometry
ICP-OES	Inductively coupled plasma-optical emission spectroscopy
LAR	Leucoanthocyanidin reductase
LOX	Lipoxygenase
OAV	Odor activity value
PCA	Principal component analysis
PDA	Photodiode array
PLS-DA	Partial least squares discriminant analysis
POD	Peroxidase
PPO	Polyphenol oxidase
QDA	Sensory evaluation and quantitative descriptive analysis
TPS	Terpene synthase
UGT	Uridine diphosphate–dependent glycosyltransferase
UHPLC-Q Exactive-Orbitrap MS	Ultra-high-performance liquid chromatography system coupled to a Q Exactive-Orbitrap mass spectrometer
UPLC	Ultra-performance liquid chromatography
VOCs	Volatile organic compounds
VIP	Variable importance in projection
ZHDB	Zhenghe Dabai
