# Supplementary material for: Chemical Drivers of Flavor Variation Across Cultivars and Grades of Fujian White Tea Revealed by Integrated Volatile and Non-Volatile Metabolomics
Source: Foods. 2026 Jan 28;15(3):458. doi: 10.3390/foods15030458 (PMC12896726; doi:10.3390/foods15030458)
Supplement: Supplementary file 1 [file foods-15-00458-s001.zip › foods-4089432-supplementary.pdf]

Section S1. Detailed List of Specific Chemicals and Reagents

Certified reference standards for catechins and related polyphenols were obtained as follows. Epicatechin (EC), gallic acid (GA), and caffeine were purchased from Shanghai Yuanye Bio-Technology Co., Ltd. Epigallocatechin (EGC), epicatechin gallate (ECG), epigallocatechin gallate (EGCG), gallocatechin gallate (GCG), and catechin (C) were purchased from Anpu Experimental Reagent Co., Ltd. (Shanghai, China). Theaflavin (TF), theaflavin-3-gallate (TF-3-G), theaflavin-3′-gallate (TF-3′-G), and theaflavin-3,3′-digallate (TFDG) were obtained from Anpu Experimental Reagent Co., Ltd. Theanine was purchased from Anpu Experimental Reagent Co., Ltd. Total flavonoids assay reagents were obtained from Shanghai Yuanye Bio-Technology Co., Ltd.

LC-MS grade acetonitrile and formic acid were obtained from Merck (Darmstadt, Germany) and Sigma-Aldrich (Steinheim, Germany), respectively. high-performance liquid chromatography (HPLC)-grade methanol and general analytical reagents for colorimetric and physicochemical analyses, including Folin-Ciocalteu phenol reagent, Coomassie Brilliant Blue G-250, o-phthalaldehyde, aluminum chloride ($AlCl_3$), sodium nitrite ($NaNO_2$), sodium hydroxide (NaOH), sodium carbonate (Na_2CO_3), ninhydrin, ferrous sulfate heptahydrate, potassium sodium tartrate tetrahydrate, potassium dihydrogen phosphate, and disodium hydrogen phosphate dodecahydrate, were purchased from Sinopharm Chemical Reagent Co., Ltd. (Shanghai, China), Wenzhou Jixiang Chemical Co., Ltd., Guangdong Guanghua Sci-Tech Co., Ltd., and Yonghua Chemical Co., Ltd.

Reagents for protein determination using the Kjeldahl method, including sulfuric acid, sodium hydroxide, boric acid, and a copper catalyst, were supplied by Zhejiang Evergreen Chemical Co., Ltd., Shanghai Titan Scientific Co., Ltd., and Fosswa Beijing Technology Co., Ltd. Trace-metal-grade nitric acid (HNO_3) was used for the wet digestion of tea samples before Inductively coupled plasma-optical emission spectroscopy (ICP-OES) analysis. A C_7-C_{40} n-alkanes mixture used for calculating linear retention indices was obtained from BePure Technology Co., Ltd. (Shanghai, China).

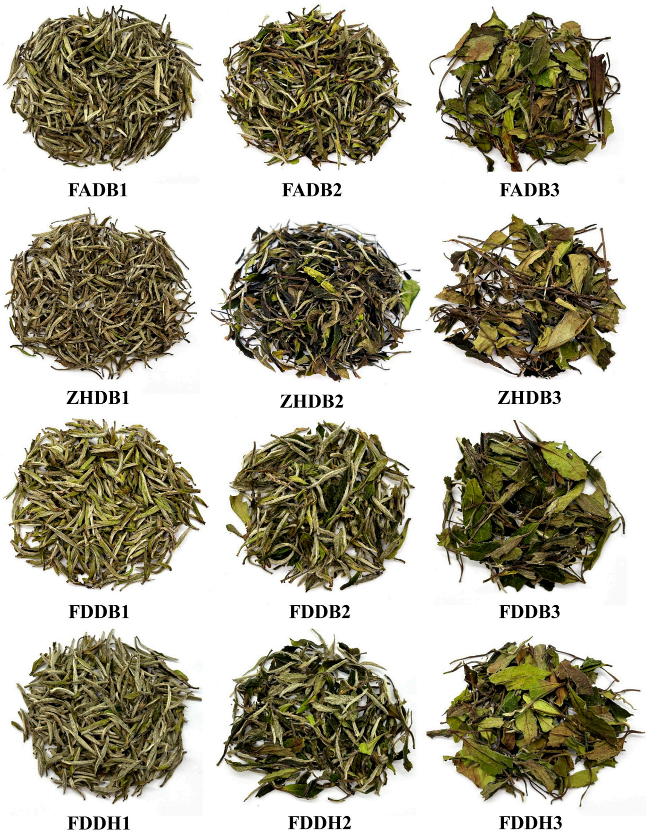

Figure S1. Representative appearance of dry white tea samples classified by cultivar and grade.

Table S1. Information and abbreviations of the twelve white tea samples categorized by cultivar and grade.

No.	Abbreviation	Cultivar	Grade	Year	Tea Varieties
**1**	**FADB1**	Fuan Dabai	Grade 1	2024	*Camellia sinensis* (L.) Kuntze cv. Fuan-dabaicha
**2**	**FADB2**	Fuan Dabai	Grade 2	2024	*Camellia sinensis* (L.) Kuntze cv. Fuan-dabaicha
**3**	**FADB3**	Fuan Dabai	Grade 3	2024	*Camellia sinensis* (L.) Kuntze cv. Fuan-dabaicha
**4**	**ZHDB1**	Zhenghe Dabai	Grade 1	2024	*Camellia sinensis* (L.) Kuntze cv. Zhenghe-dabaicha
**5**	**ZHDB2**	Zhenghe Dabai	Grade 2	2024	*Camellia sinensis* (L.) Kuntze cv. Zhenghe-dabaicha
**6**	**ZHDB3**	Zhenghe Dabai	Grade 3	2024	*Camellia sinensis* (L.) Kuntze cv. Zhenghe-dabaicha
**7**	**FDDB1**	Fuding Dabai	Grade 1	2024	*Camellia sinensis* (L.) Kuntze cv. Fuding-dabaicha
**8**	**FDDB2**	Fuding Dabai	Grade 2	2024	*Camellia sinensis* (L.) Kuntze cv. Fuding-dabaicha
**9**	**FDDB3**	Fuding Dabai	Grade 3	2024	*Camellia sinensis* (L.) Kuntze cv. Fuding-dabaicha
**10**	**FDDH1**	Fuding Dahao	Grade 1	2024	*Camellia sinensis* (L.) Kuntze cv. Fuding-dahaocha
**11**	**FDDH2**	Fuding Dahao	Grade 2	2024	*Camellia sinensis* (L.) Kuntze cv. Fuding-dahaocha
**12**	**FDDH3**	Fuding Dahao	Grade 3	2024	*Camellia sinensis* (L.) Kuntze cv. Fuding-dahaocha

All samples were harvested in April 2024.

Table S2. Physicochemical parameters and analytical methods used for white tea quality evaluation.

**Parameter**	**Analytical method**	**Standard or reference**
Total polyphenols	Folin–Ciocalteu colorimetric assay	GB/T 8313
Protein	Coomassie Brilliant Blue G-250 assay	GB/T 5009.5
Amino acid nitrogen	Ninhydrin colorimetry	GB 5009.235-2016
Free amino acids	Ninhydrin colorimetry	GB/T 8314-2013
Moisture content	Oven drying (105 °C to constant weight)	GB/T 8304
Theanine	OPA pre-column derivatization–HPLC (FLD)	GB/T 23193-2017
Theaflavin	HPLC	GB/T 30483-2013
Theaflavin-3-gallate	HPLC	GB/T 30483-2013
Theaflavin-3′-gallate	HPLC	GB/T 30483-2013
Theaflavin-3,3′-digallate	HPLC	GB/T 30483-2013
Total flavonoids	$AlCl_3$ colorimetric assay	QB/T 5206-2019
Mineral elements (Ca, K, Mg, Mn, Fe, Zn, Al)	ICP–OES after HNO_3 digestion	GB 5009 series*

* Ca: GB 5009.92-2016; K: GB 5009.91-2017; Mg: GB 5009.241-2017; Mn: GB 5009.242-2017; Fe: GB 5009.90-2016; Zn: GB 5009.14-2017; Al: GB 5009.268-2025.

Table S3. Key non-volatile metabolites (VIP ≥ 1.5) discriminating different grades of white tea.

No.	VIP	RT [min]	Accurate [M-H]$^-$	Metabolomics identification	Categories	Molecular formula	MS2 fragments
1	12.76	5.52	457.0779	Epigallocatechin gallate	Flavan-3-ols	$C_{22}H_{18}O_{11}$	305, 225, 169, 125
2	11.38	2.29	343.0670	Theogallin	Phenolic acids	$C_{14}H_{16}O_{10}$	245, 191, 169, 125
3	9.11	6.44	441.0829	Epicatechin gallate	Flavan-3-ols	$C_{22}H_{18}O_{10}$	289, 169, 125
4	9.05	4.58	305.0666	Epigallocatechin	Flavan-3-ols	$C_{15}H_{14}O_7$	261, 219, 167, 125
5	9.04	1.07	191.0552	Quinic acid	Organic acids	$C_7H_{12}O_6$	173, 127, 85
6	6.72	6.29	609.0890	Epigallocatechin 3,5-di-*O*-gallate	Flavan-3-ols	$C_{29}H_{22}O_{15}$	457, 305, 169
7	6.70	5.10	577.1355	Procyanidin B2	Dimeric catechins	$C_{30}H_{26}O_{12}$	425, 407, 289, 161
8	6.27	5.63	635.0892	Trigalloyl glucose	Phenolic acids	$C_{27}H_{24}O_{18}$	483, 465, 313, 169
9	5.50	6.92	447.0936	Kaempferol-3-*O*-galactoside	Flavonol *O*-glycosides	$C_{21}H_{20}O_{11}$	285, 255, 227, 151
10	5.42	5.13	913.1479	Theasinensin A	Dimeric catechins	$C_{44}H_{34}O_{22}$	457, 305, 169, 125
11	5.42	4.64	483.0781	Digalloylglucose	Phenolic acids	$C_{20}H_{20}O_{14}$	423, 331, 271, 169
12	5.35	6.04	771.1996	Quercetin 3-*O*-glucosylrutinoside	Flavonol *O*-glycosides	$C_{33}H_{40}O_{21}$	609, 463, 301, 179
13	4.85	2.21	169.0134	Gallic acid	Phenolic acids	$C_7H_6O_5$	125, 79
14	4.71	7.09	425.0881	Epiafzelechin 3-*O*-gallate	Flavan-3-ols	$C_{22}H_{18}O_9$	271, 255, 169, 125
15	3.93	1.86	331.0672	Galloylglucose	Phenolic acids	$C_{13}H_{16}O_{10}$	271, 169
16	3.88	1.10	191.0189	Citric acid	Organic acids	$C_6H_8O_7$	173, 129, 111, 87
17	3.86	5.94	479.0833	Myricetin 3-galactoside	Flavonol *O*-glycosides	$C_{21}H_{20}O_{13}$	317, 193, 169
18	3.82	1.06	383.1196	6-*O*-acetyl-maltose	Carbohydrates	$C_{14}H_{24}O_{12}$	341, 323, 179
19	3.81	1.50	173.0923	Theanine	Amino acids	$C_7H_{14}N_2O_3$	155, 128
20	3.27	12.14	293.2123	13-KODE	Oxylipins	$C_{18}H_{30}O_3$	275, 249, 223, 201
21	2.86	6.30	755.2046	Kaempferol 3-*O*-galactosylrutinoside	Flavonol *O*-glycosides	$C_{33}H_{40}O_{20}$	609, 447, 285, 255
22	2.85	12.47	295.2277	13-hydroxy-octadecadienoic acid	Oxylipins	$C_{18}H_{32}O_3$	277, 251, 233, 195
23	2.84	4.53	631.0945	Myricetin-3-*O*-galloylhexoside	Flavonol *O*-glycosides	$C_{28}H_{24}O_{17}$	479, 317, 169
24	2.81	5.47	289.0718	Catechin	Flavan-3-ols	$C_{15}H_{14}O_6$	245, 203, 125, 109
25	2.70	8.17	867.1426	Theaflavin 3,3′-digallate	Dimeric catechins	$C_{43}H_{32}O_{20}$	743, 591, 423, 169, 125

26	2.54	5.71	441.0826	Catechin gallate	Flavan-3-ols	$C_{22}H_{18}O_{10}$	289, 169, 125
27	2.50	1.05	341.1089	Maltose	Carbohydrates	$C_{12}H_{22}O_{11}$	327, 311, 309
28	2.41	6.26	609.1455	Rutin	Flavonol *O*-glycosides	$C_{27}H_{30}O_{16}$	301, 271, 255, 179
29	2.30	1.03	135.0290	L-threonic acid	Organic acids	$C_4H_8O_5$	135, 117, 89
30	2.17	1.11	133.0131	Malic acid	Organic acids	$C_4H_6O_5$	115, 89, 71
31	2.14	6.03	415.1613	2-Phenylethyl *β*-primeveroside	Aromatic glycosides	$C_{19}H_{28}O_{10}$	267, 249, 179, 161
32	2.08	6.50	463.0883	Quercetin-3*β*-D-glucoside	Flavonol *O*-glycosides	$C_{21}H_{20}O_{12}$	301, 169, 125
33	2.08	1.02	195.0503	Gluconic acid	Organic acids	$C_6H_{12}O_7$	177, 129, 75
34	1.98	5.44	337.0930	3-*O*-*p*-Coumaroylquinic acid	Phenolic acids	$C_{16}H_{18}O_8$	191, 173, 119
35	1.96	4.20	321.0615	leucodelphinidin	Leucoanthocyanidins	$C_{15}H_{14}O_8$	303, 289, 179, 151
36	1.95	6.41	300.9988	Ellagic acid	Phenolic acids	$C_{14}H_6O_8$	283, 259, 233, 199, 125
37	1.88	7.39	187.0968	Azelaic acid	Oxylipins	$C_9H_{16}O_4$	169, 125, 111, 97
38	1.73	9.46	285.0405	Kaempferol	Flavonols	$C_{15}H_{10}O_6$	285, 257, 229
39	1.70	4.81	175.0603	Isopropylmalic acid	Organic acids	$C_7H_{12}O_5$	115, 73
40	1.67	3.62	305.0666	Gallocatechin	Flavan-3-ols	$C_{15}H_{14}O_7$	261, 219, 125
41	1.66	4.51	593.1306	6″-*O*-*trans*-*p*-Coumaroyl-astragalin	Flavonol *O*-glycosides	$C_{30}H_{26}O_{13}$	447, 285, 163, 119
42	1.63	7.54	287.0563	Eriodictyol	Flavanones	$C_{15}H_{12}O_6$	151, 135, 107
43	1.60	1.03	165.0396	Arabinic acid	Organic acids	$C_5H_{10}O_6$	133, 87
44	1.55	5.10	633.0727	Strictinin	Phenolic acids	$C_{27}H_{22}O_{18}$	463, 301, 169, 125
45	1.54	1.91	609.1256	Prodelphinidin B4	Dimeric catechins	$C_{30}H_{26}O_{14}$	451, 305, 289, 169
46	1.52	2.85	164.0709	Phenylalanine	Amino acids	$C_9H_{11}NO_2$	164, 147, 120
47	1.50	8.46	301.0355	Quercetin	Flavonols	$C_{15}H_{10}O_7$	179, 151

Metabolites were identified by MS/MS spectral matching using Compound Discoverer 3.2.0 integrated with the mzCloud and ChemSpider databases.

Table S4. Key non-volatile metabolites (VIP ≥ 1.5) discriminating different cultivars of white tea.

No.	VIP	RT [min]	Accurate [M-H]$^-$	Metabolomics identification	Categories	Molecular formula	MS2 fragments
1	10.57	6.29	609.0890	Epigallocatechin 3,5-di-*O*-gallate	Flavan-3-ols	$C_{29}H_{22}O_{15}$	457, 305, 169
2	9.32	4.58	305.0666	Epigallocatechin	Flavan-3-ols	$C_{15}H_{14}O_7$	261, 219, 167, 125
3	8.30	5.52	457.0779	Epigallocatechin gallate	Flavan-3-ols	$C_{22}H_{18}O_{11}$	305, 225, 169, 125
4	7.40	5.10	577.1355	Procyanidin B2	Dimeric catechins	$C_{30}H_{26}O_{12}$	425, 407, 289, 161
5	7.35	1.07	191.0552	Quinic acid	Organic acids	$C_7H_{12}O_6$	173, 127, 85
6	6.11	1.10	191.0189	Citric acid	Organic acids	$C_6H_8O_7$	173, 129, 111, 87
7	5.99	7.09	425.0881	Epiafzelechin 3-*O*-gallate	Flavan-3-ols	$C_{22}H_{18}O_9$	271, 255, 169, 125
8	5.99	1.07	209.0658	*α*-D-manno-heptulopyranose	Carbohydrates	$C_7H_{14}O_7$	191, 179, 161
9	5.98	7.04	455.0986	3′-*O*-methyl-Epicatechin gallate	Flavan-3-ols	$C_{23}H_{20}O_{10}$	221, 161, 109, 97
10	5.88	6.44	441.0829	Epicatechin gallate	Flavan-3-ols	$C_{22}H_{18}O_{10}$	289, 169, 125
11	5.64	5.63	635.0892	Trigalloyl glucose	Phenolic acids	$C_{27}H_{24}O_{18}$	483, 465, 313, 169
12	5.48	3.62	305.0666	Gallocatechin	Flavan-3-ols	$C_{15}H_{14}O_7$	261, 219, 125
13	5.45	12.14	293.2123	13-KODE	Oxylipins	$C_{18}H_{30}O_3$	275, 249, 223, 201
14	4.62	12.47	295.2277	13-hydroxy-octadecadienoic acid	Oxylipins	$C_{18}H_{32}O_3$	277, 251, 233, 195
15	4.60	5.94	479.0833	Myricetin 3-galactoside	Flavonol *O*-glycosides	$C_{21}H_{20}O_{13}$	317, 193, 169
16	4.21	1.06	383.1196	6-*O*-acetyl-maltose	Carbohydrates	$C_{14}H_{24}O_{12}$	341, 323, 179
17	3.95	1.50	173.0923	Theanine	Amino acids	$C_7H_{14}N_2O_3$	155, 128
18	3.75	2.21	169.0134	Gallic acid	Phenolic acids	$C_7H_6O_5$	125, 79
19	3.36	4.51	593.1306	6″-*O*-*trans*-*p*-Coumaroyl-astragalin	Flavonol *O*-glycosides	$C_{30}H_{26}O_{13}$	447, 285, 163, 119
20	3.23	1.86	331.0672	Galloylglucose	Phenolic acids	$C_{13}H_{16}O_{10}$	271, 169
21	3.10	3.93	609.1256	Prodelphinidin B2	Dimeric catechins	$C_{30}H_{26}O_{14}$	423, 407, 305, 289
22	3.03	4.64	483.0781	Digalloylglucose	Phenolic acids	$C_{20}H_{20}O_{14}$	423, 331, 271, 169
23	2.82	6.92	447.0936	Kaempferol-3-*O*-galactoside	Flavonol *O*-glycosides	$C_{21}H_{20}O_{11}$	285, 255, 227, 151
24	2.76	6.72	467.0986	Epigallocatechin 3-*O*-caffeate	Flavan-3-ols	$C_{24}H_{20}O_{10}$	305, 179, 161

25	2.74	1.02	195.0503	Gluconic acid	Organic acids	$C_6H_{12}O_7$	177, 129, 75
26	2.74	6.26	609.1455	Rutin	Flavonol *O*-glycosides	$C_{27}H_{30}O_{16}$	301, 271, 255, 179
27	2.66	1.03	135.0290	L-threonic acid	Organic acids	$C_4H_8O_5$	135, 117, 89
28	2.58	5.47	289.0718	Catechin	Flavan-3-ols	$C_{15}H_{14}O_6$	245, 203, 125, 109
29	2.43	1.03	165.0396	Arabinic acid	Organic acids	$C_5H_{10}O_6$	133, 87
30	2.35	5.44	337.0930	3-*O*-*p*-Coumaroylquinic acid	Phenolic acids	$C_{16}H_{18}O_8$	191, 173, 119
31	2.27	6.03	415.1613	2-Phenylethyl *β*-primeveroside	Aromatic glycosides	$C_{19}H_{28}O_{10}$	253, 179, 161, 121
32	2.02	11.85	311.2229	(10E,12Z)-9-Hydroperoxy-10,12-octadecadienoic acid	Oxylipins	$C_{18}H_{32}O_4$	295, 223, 195, 171
33	2.01	4.81	175.0603	Isopropylmalic acid	Organic acids	$C_7H_{12}O_5$	115, 73
34	1.97	6.41	300.9988	Ellagic acid	Phenolic acids	$C_{14}H_6O_8$	283, 259, 233, 199, 125
35	1.96	9.46	285.0405	Kaempferol	Flavonols	$C_{15}H_{10}O_6$	285, 257, 229
36	1.88	6.50	463.0883	Quercetin-3*β*-D-glucoside	Flavonol *O*-glycosides	$C_{21}H_{20}O_{12}$	301, 169, 125
37	1.81	4.92	353.0877	Chlorogenic acid	Phenolic acids	$C_{16}H_{18}O_9$	191. 179. 135
38	1.80	14.03	279.2329	Linoleic acid	Oxylipins	$C_{18}H_{32}O_2$	261, 233, 171
39	1.80	5.82	625.1416	Myricetin 3-rutinoside	Flavonol *O*-glycosides	$C_{27}H_{30}O_{17}$	463, 449, 317, 179
40	1.77	4.20	321.0615	Leucodelphinidin	Leucoanthocyanidins	$C_{15}H_{14}O_8$	303, 289, 179, 151
41	1.71	13.54	277.2173	*α*-Linolenic acid	Oxylipins	$C_{18}H_{30}O_2$	259, 233, 171
42	1.59	13.66	271.2280	Juniperic acid	Oxylipins	$C_{16}H_{32}O_3$	253, 227, 183
43	1.59	5.29	865.1998	Procyanidin C1	Dimeric catechins	$C_{45}H_{38}O_{18}$	739, 577, 289
44	1.51	6.15	393.1768	cis-3-Hexenyl -primeveroside	Aromatic glycosides	$C_{17}H_{30}O_{10}$	231, 179, 99

Metabolites were identified by MS/MS spectral matching using Compound Discoverer 3.2.0 integrated with the mzCloud and ChemSpider databases.

Table S5. Volatile compounds with OAVs in samples exceeded 1.

Compounds	Odor description	OT (μg/L)	FADB1	FADB2	FADB3	ZHDB1	ZHDB2	ZHDB3	FDDB1	FDDB2	FDDB3	FDDH1	FDDH2	FDDH3
Linalool	Floral aroma	0.6	119	106	86	82	75	50	124	290	240	200	261	138
Geraniol	Floral aroma	3.2	88	65	64	113	89	18	10	19	16	19	19	8
(E,Z)-3,6-Nonadien-1-ol	Green aroma	0.003	75	63	45	80	40	39	123	338	75	213	155	59
(E)-Citral	Fruity aroma	0.1	8	5	3	6	4	< 1	< 1	1	< 1	1	< 1	< 1
2-Octanone	Fruity aroma	0.05	7	11	8	14	12	5	16	9	9	11	9	7
1-Octanol	Fruity aroma	0.13	3	6	11	4	7	10	6	14	8	5	8	10
1-Hexanol	Green aroma	0.5	3	4	5	3	3	5	7	9	6	7	6	6
3,7-Dimethyl-1,5,7-octatrien-3-ol	Floral aroma	0.245	2	5	5	3	8	6	5	19	9	4	8	6
Geranyl acetate	Floral aroma	0.15	2	1	1	2	n.d.	n.d.	n.d.	n.d.	n.d.	n.d.	n.d.	n.d.
(Z)-3,7-Dimethyl-2,6-octadien-1-ol	Floral aroma	2.2	1	1	1	1	1	1	0	1	1	0	1	0
1-Nonanol	Sweet aroma	1	1	2	3	1	1	1	1	4	1	1	1	1
(Z)-Jasmone	Floral aroma	0.26	1	1	1	0	n.d.	n.d.	1	1	1	1	n.d.	n.d.
(Z)-3-Hexen-1-ol	Green aroma	3.9	1	1	2	1	1	1	3	3	3	2	2	3

p-Cymene	Herbal aroma	0.15	n.d.	1	n.d.	1	1	n.d.	n.d.	1	n.d.	n.d.	n.d.	n.d.

Note: OAV, odor activity value.

n.d., not detected.

Table S6. Volatile compounds of white tea from different samples.

NO.	Compounds	CAS	RI[a]	Contents (mg/L)											
				FADB1	FADB2	FADB3	ZHDB1	ZHDB2	ZHDB3	FDDB1	FDDB2	FDDB3	FDDH1	FDDH2	FDDH3
1	Hexanal	66-25-1	1083	1.26±0.15a	0.97±0.08b	0.65±0.05cd	0.73±0.10c	0.78±0.10c	0.51±0.05d	0.89±0.09b	0.56±0.07d	0.33±0.06e	0.87±0.15b	0.56±0.06d	0.74±0.10c
2	2-Heptanone	110-43-0	1184	0.83±0.10de	1.37±0.08c	0.95±0.10de	1.56±0.23bc	1.77±0.12b	0.76±0.03e	2.21±0.25a	1.05±0.08d	0.74±0.15e	1.38±0.25c	0.93±0.08de	0.49±0.07f
3	(*E*)-2-Hexenal	6728-26-3	1216	3.01±0.25c	3.51±0.26b	3.58±0.57b	3.57±0.38b	3.45±0.36b	2.01±0.08d	2.90±0.38c	2.67±0.31c	4.41±0.46a	3.38±0.08b	3.41±0.28b	3.70±0.31b
4	1-Pentanol	71-41-0	1258	0.34±0.02c	0.34±0.04c	0.15±0.03f	0.33±0.05cd	0.29±0.02d	0.20±0.02e	0.72±0.02a	0.56±0.03b	0.15±0.00f	0.54±0.03b	0.31±0.01cd	0.13±0.01f
5	*p*-Cymene	99-87-6	1263	n.d.	0.11±0.01b	n.d.	0.18±0.02a	0.09±0.01c	n.d.	n.d.	0.12±0.02b	n.d.	n.d.	n.d.	n.d.
6	2-Octanone	111-13-7	1285	0.33±0.04de	0.56±0.05c	0.39±0.03d	0.70±0.08b	0.62±0.04b	0.27±0.01e	0.82±0.16a	0.43±0.03d	0.46±0.02c	0.55±0.08c	0.45±0.06c	0.32±0.00de
7	2-Ethyl-2-hexenal	645-62-5	1333	0.33±0.04d	0.19±0.02f	n.d.	0.39±0.06c	0.14±0.01g	0.11±0.00g	0.83±0.03a	0.27±0.02e	n.d.	0.55±0.03b	0.15±0.01fg	n.d.
8	5-Hepten-2-one, 6-methyl-	110-93-0	1339	3.26±0.17b	2.50±0.41cd	2.15±0.24d	3.95±0.49a	2.55±0.23c	0.98±0.03f	1.49±0.07e	1.01±0.07f	1.13±0.06f	1.66±0.03e	1.57±0.12e	1.43±0.11e
9	1-Hexanol	111-27-3	1358	1.24±0.11f	1.83±0.11e	2.36±0.36d	1.53±0.19ef	1.32±0.22f	2.42±0.32d	3.47±0.18b	4.43±0.17a	2.73±0.14c	3.34±0.07b	3.20±0.07b	2.78±0.15c
10	(*Z*)-3-Hexen-1-ol	928-96-1	1388	1.91±0.15f	3.06±0.21e	6.55±0.78d	2.61±0.30ef	2.98±0.49e	2.05±0.39f	10.22±0.86b	11.45±0.40a	11.09±1.07a	9.44±0.20b	7.61±0.22c	10.05±0.49b
11	2-Nonanone	821-55-6	1391	0.30±0.06c	0.48±0.03b	0.42±0.04b	0.61±0.09a	0.54±0.05a	0.25±0.03c	0.56±0.10a	0.42±0.04b	0.44±0.09b	0.42±0.01b	0.43±0.02b	0.40±0.06b
12	3-Octen-2-one	1669-44-9	1409	0.72±0.08c	0.54±0.08d	0.37±0.05e	0.42±0.06e	0.48±0.03d	0.50±0.06d	1.12±0.04a	1.18±0.07a	0.36±0.06e	0.85±0.07b	0.43±0.04e	0.16±0.03f
13	(*E*)-2-Hexen-1-ol	928-95-0	1410	0.21±0.02f	0.50±0.07d	1.14±0.14a	0.25±0.04f	0.42±0.07de	0.63±0.12c	0.46±0.02de	0.85±0.06b	0.62±0.05c	0.37±0.02e	0.48±0.03de	0.71±0.05c
14	(*Z*)-Linalool oxide (furanoid)	5989-33-3	1443	6.13±0.62d	8.94±0.41b	12.09±1.08a	5.17±0.79e	7.58±0.23c	4.79±0.32e	1.80±0.08f	7.07±0.78cd	6.79±0.25cd	2.29±0.12f	6.11±0.24d	6.20±0.16d
15	1-Octen-3-ol	3391-86-4	1456	0.41±0.01de	0.54±0.04c	0.46±0.05d	0.49±0.04d	0.55±0.04c	0.35±0.03e	1.03±0.13a	0.82±0.05b	0.60±0.01c	0.88±0.02b	0.59±0.03c	0.45±0.04d

16	1-Heptanol	111-70-6	1459	1.08±0.11d	0.84±0.11d	1.45±0.24c	0.87±0.15d	1.03±0.18d	0.84±0.10d	1.80±0.14b	2.63±0.14a	1.21±0.04c	1.34±0.09c	1.31±0.04c	1.20±0.20c
17	(*R,S*)-5-Ethyl-6-methyl-3*E*-hepten-2-one	57283-79-1	1465	2.74±0.35c	2.67±0.34c	1.88±0.25d	3.22±0.50b	2.50±0.14c	1.34±0.16e	5.06±0.30a	3.15±0.16b	1.98±0.04d	3.25±0.22b	2.43±0.10c	1.00±0.12e
18	6-Methyl-5-hepten-2-ol	1569-60-4	1468	0.17±0.03e	0.27±0.05d	0.47±0.08bc	0.44±0.07bc	0.39±0.04c	0.62±0.05a	0.53±0.08b	0.64±0.07a	0.37±0.02c	0.44±0.03bc	0.41±0.01c	0.65±0.07a
19	*trans*-Linalool oxide (furanoid)	34995-77-2	1472	27.60±3.25b	29.91±4.71b	36.27±2.67a	11.05±1.57d	16.50±0.82c	8.86±0.66e	3.10±0.25f	14.37±0.85cd	14.01±1.14cd	5.76±0.33ef	13.75±0.24cd	12.79±0.24d
20	2-Ethyl-hexanol-1	104-76-7	1494	0.15±0.03a	n.d.	0.12±0.01bc	n.d.	n.d.	n.d.	n.d.	n.d.	0.12±0.01bc	0.11±0.01c	0.13±0.01bc	0.13±0.01b
21	(*E,E*)-2,4-Heptadienal	4313-03-5	1496	0.38±0.05c	0.42±0.06c	0.58±0.11b	0.17±0.03de	0.45±0.07c	1.31±0.07a	0.19±0.02de	0.22±0.01d	0.11±0.01e	n.d.	n.d.	0.12±0.01e
22	Benzaldehyde	100-52-7	1524	16.70±2.48b	15.37±2.63b	14.07±2.09bc	19.64±2.82a	21.08±1.49a	12.17±1.04cd	13.18±0.89c	12.86±2.41c	11.65±0.91cd	11.35±1.15cd	9.51±0.48d	6.41±0.87e
23	6-Undecanone	927-49-1	1530	n.d.	0.13±0.01c	n.d.	0.17±0.02b	n.d.	n.d.	0.19±0.02a	0.12±0.01cd	n.d.	0.11±0.01d	n.d.	n.d.
24	Linalool	78-70-6	1554	71.49±11.97f	63.84±6.88f	51.44±2.32g	49.34±6.30g	44.76±2.34g	30.01±3.74h	74.18±4.34e	174.13±8.66a	143.96±1.73c	120.08±2.90d	156.74±4.85b	82.53±4.09e
25	1-Octanol	111-87-5	1562	0.43±0.07e	0.74±0.12d	1.45±0.22b	0.45±0.07e	0.92±0.11c	1.33±0.21b	0.75±0.13d	1.85±0.28a	1.07±0.01c	0.59±0.06de	1.04±0.08c	1.30±0.24b
26	6-Methyl-3,5-heptadiene-2-one	1604-28-0	1595	0.42±0.06c	0.46±0.07c	0.70±0.12b	0.62±0.08b	0.72±0.10ab	0.45±0.06c	0.63±0.04b	0.44±0.04c	0.63±0.03b	0.49±0.01c	0.64±0.06b	0.84±0.12a
27	3,7-Dimethyl-1,5,7-octatrien-3-ol	29957-43-5	1615	0.59±0.06d	1.24±0.19c	1.20±0.07c	0.79±0.14d	1.96±0.13b	1.38±0.12c	1.22±0.14c	4.67±0.78a	2.28±0.05b	0.97±0.14cd	1.97±0.08b	1.37±0.15c
28	*β*-Cyclocitral	432-25-7	1619	0.14±0.01e	0.24±0.04d	0.51±0.10a	0.15±0.02e	0.32±0.04c	0.57±0.05a	0.15±0.01e	0.28±0.00cd	0.39±0.01b	0.24±0.02d	0.33±0.01c	0.42±0.02b
29	Acetophenone	98-86-2	1652	0.21±0.03e	0.22±0.02e	0.31±0.06d	0.37±0.04c	0.29±0.03d	0.28±0.00d	0.45±0.03b	0.25±0.02e	0.32±0.02c	0.54±0.03a	0.37±0.00c	0.26±0.03de

30	1-Nonanol	143-08-8	1664	0.66±0.07e	1.72±0.11c	2.58±0.48b	1.19±0.21d	1.45±0.22c	0.98±0.15de	1.33±0.19c	4.21±0.63a	1.41±0.03c	0.80±0.09de	1.40±0.13c	1.18±0.18d
31	Neral	106-26-3	1683	0.36±0.04a	0.25±0.03b	0.14±0.01c	0.26±0.05b	0.14±0.02c	n.d.	n.d.	n.d.	n.d.	n.d.	n.d.	n.d.
32	(*Z*)-3-Nonen-1-ol	10340-23-5	1687	0.32±0.03f	0.36±0.06ef	0.37±0.07ef	0.46±0.05e	0.30±0.04f	0.35±0.06ef	0.58±0.08d	1.52±0.12a	0.42±0.04e	0.91±0.01b	0.77±0.04c	0.34±0.04f
33	2,6,6-Trimethyl-2-cyclohexen-1,4-dione	1125-21-9	1693	n.d.	0.13±0.02b	0.11±0.01c	0.13±0.02b	0.11±0.00c	n.d.	0.17±0.01a	0.12±0.01bc	0.12±0.01bc	0.11±0.00c	0.11±0.01c	n.d.
34	*α*-Terpineol	98-55-5	1699	2.15±0.23b	2.38±0.44b	1.63±0.22c	0.58±0.08de	4.04±0.63a	0.19±0.03e	0.45±0.04de	0.72±0.03d	0.75±0.03d	0.54±0.02de	0.75±0.04d	0.63±0.04de
35	(*E*)-Citral	141-27-5	1732	0.76±0.10a	0.45±0.09c	0.32±0.04d	0.58±0.09b	0.36±0.06d	n.d.	n.d.	0.11±0.01e	n.d.	0.11±0.00e	n.d.	n.d.
36	(*E*,*Z*)-3,6-Nonadien-1-ol	56805-23-3	1751	0.22±0.02e	0.19±0.02e	0.13±0.03fg	0.24±0.02e	0.12±0.01g	0.12±0.01g	0.37±0.04d	1.01±0.08a	0.23±0.02e	0.64±0.03b	0.47±0.01c	0.18±0.02f
37	*δ*-Cadinene	483-76-1	1752	0.70±0.10a	0.54±0.04b	0.23±0.04c	n.d.	n.d.	n.d.	0.19±0.02cd	0.23±0.04c	n.d.	0.25±0.03c	0.13±0.02d	n.d.
38	Geranyl acetate	105-87-3	1757	0.24±0.01b	0.19±0.04c	0.20±0.03c	0.33±0.03a	n.d.	n.d.	n.d.	n.d.	n.d.	n.d.	n.d.	n.d.
39	Methyl salicylate	119-36-8	1773	68.92±10.19b	95.03±16.97a	75.92±6.94b	51.69±9.79c	42.31±4.36d	8.60±1.67f	26.58±5.31e	102.48±5.55a	50.94±3.07c	35.21±0.73de	58.50±7.71c	34.28±0.62de
40	2-Phenethyl formate	104-62-1	1787	0.09±0.02c	0.14±0.02a	n.d.	n.d.	n.d.	n.d.	n.d.	0.12±0.01b	n.d.	0.12±0.02b	n.d.	n.d.
41	(*Z*)-3,7-Dimethyl-2,6-octadien-1-ol	106-25-2	1802	2.51±0.31b	2.28±0.24b	3.09±0.60a	3.18±0.42a	2.88±0.41a	1.08±0.19c	0.52±0.09d	1.07±0.05c	1.01±0.10cd	0.84±0.04cd	1.10±0.06c	0.89±0.11cd
42	Hexanoic acid	142-62-1	1843	3.44±0.60c	5.52±0.25b	4.31±0.47c	6.07±0.86b	5.42±0.66b	1.78±0.35d	10.49±1.40a	3.85±0.51c	5.47±0.12b	5.00±0.68b	3.27±0.52c	1.76±0.33d
43	Geraniol	106-24-1	1847	282.14±30.87b	207.07±21.30c	204.72±38.77c	361.00±57.64a	283.10±46.94b	57.13±8.60d	31.71±6.12d	59.91±4.93d	50.69±4.20d	61.18±2.75d	59.47±2.93d	24.20±3.58d
44	3-Phenylfuran	13679-41-9	1850	n.d.	0.13±0.02b	0.14±0.02a	0.14±0.02a	0.16±0.02a	0.13±0.01b	n.d.	0.12±0.01b	0.11±0.01b	0.11±0.01b	0.14±0.01a	n.d.

45	(*E*)-6,10-Dimethyl-5,9-undecadien-2-one	3796-70-1	1851	1.09±0.13bc	1.00±0.14c	1.90±0.28a	1.31±0.25b	1.37±0.26b	1.12±0.13bc	0.88±0.10c	1.13±0.04bc	0.86±0.04c	0.87±0.06c	1.13±0.02bc	1.81±0.35a
46	Benzyl alcohol	100-51-6	1873	7.29±0.69e	6.77±1.02ef	6.31±1.02ef	9.00±1.17d	8.13±1.38d	5.41±0.76f	12.52±0.75b	15.91±1.26a	10.85±0.69c	15.68±0.33a	15.00±0.93a	10.55±0.76c
47	*N*-ethylsuccinimide	2314-78-5	1876	n.d.	0.16±0.03c	0.26±0.05b	0.14±0.02c	0.24±0.02b	0.43±0.07a	0.21±0.03b	n.d.	0.27±0.02b	0.11±0.01c	0.17±0.03c	0.23±0.04b
48	Phenylethyl Alcohol	60-12-8	1908	32.95±3.30d	25.19±2.78e	17.92±3.11f	22.10±2.68f	13.08±1.83g	12.67±0.89g	62.48±1.69a	50.21±5.82b	33.61±2.01d	63.86±2.53a	42.73±2.02c	28.41±3.54e
49	(*Z*)-Jasmone	488-10-8	1931	0.14±0.03cd	0.15±0.02c	0.22±0.04b	0.11±0.01d	n.d.	n.d.	0.33±0.03a	0.21±0.01b	0.14±0.00cd	0.21±0.02b	n.d.	n.d.
50	Heptanoic acid	111-14-8	1949	0.16±0.03de	0.36±0.05c	0.30±0.03c	0.61±0.09a	0.46±0.08b	n.d.	0.39±0.04b	0.18±0.03de	0.23±0.02d	0.20±0.02de	0.14±0.01e	n.d.
51	(*E*)-2-Hexenoic acid	13419-69-7	1963	0.55±0.09b	0.66±0.10b	0.77±0.10a	0.84±0.12a	0.83±0.15a	0.27±0.04d	0.49±0.10c	0.51±0.02b	0.52±0.09b	0.45±0.05c	0.34±0.03cd	0.30±0.05d
52	*γ*-Nonanolactone	104-61-0	2014	0.66±0.11cd	0.61±0.10cd	0.94±0.15b	0.94±0.16b	0.71±0.07c	0.80±0.09b	1.24±0.08a	0.50±0.05d	0.82±0.02b	0.78±0.07bc	0.65±0.03cd	0.50±0.07d
53	Nonanoic acid	112-05-0	2174	0.12±0.01d	0.20±0.01c	0.28±0.05b	0.38±0.06a	0.29±0.03b	n.d.	0.19±0.04c	0.16±0.01cd	0.19±0.01c	0.15±0.03cd	0.12±0.01d	n.d.
54	Dihydroactinidiolide	17092-92-1	2330	0.25±0.05d	0.28±0.04d	0.81±0.16a	0.41±0.06c	0.52±0.10c	0.53±0.07c	0.30±0.05d	0.31±0.03d	0.51±0.02c	0.31±0.03d	0.38±0.01d	0.68±0.10b
55	Geranic acid	4698-8-2	2347	n.d.	0.16±0.02b	0.21±0.04a	0.15±0.02b	0.21±0.04a	n.d.	n.d.	n.d.	n.d.	n.d.	n.d.	n.d.

Note: (a) RI: Retention indices were calculated using a homologous series of n-alkanes (C_7-C_{40}) analyzed on an HP-INNOWAX capillary column under the same GC conditions. (b) Identification criteria: compounds were identified by EI mass spectra (MS) and RI matching. (c) n.d., not detected and excluded from statistical analysis. FADB, ZHDB, FDDB, and FDDH denote four cultivars, and 1-3 indicate three grades, respectively. Values are presented as mean ± SD (n = 3). Different lowercase letters within the same row indicate significant differences among samples (one-way ANOVA followed by Duncan's multiple range test, $p < 0.05$).
